# Effect of routine suction on lung aeration in critically ill neonates and young infants measured with electrical impedance tomography

**DOI:** 10.1038/s41598-023-42965-7

**Published:** 2023-11-27

**Authors:** Claas Händel, Tobias Becher, Martijn Miedema, Merja Kallio, Thalia Papadouri, Andreas D. Waldmann, Louiza Sophocleous, Christina Karaoli, Rebecca Yerworth, Richard Bayford, Peter C. Rimensberger, Anton H. van Kaam, Inéz Frerichs

**Affiliations:** 1 Department of Anaesthesiology and Intensive Care Medicine, University Medical Center Schlewig-Holstein, Campus Kiel, Kiel, Germany; 2 Department of Neonatology, Emma Children’s Hospital, Amsterdam UMC, Amsterdam, The Netherlands; 3 Amsterdam Reproduction and Development Research Institute, Amsterdam, The Netherlands; 4 Department of Pediatrics and Adolescent Medicine, Oulu University Hospital, and Research Unit of Clinical Medicine, University of Oulu, Oulu, Finland; 5 Neonatal Intensive Care Unit, Arch. Makarios III Hospital, Nicosia, Cyprus; 6 Department of Anesthesiology and Intensive Care Medicine, University Medical Center Rostock, Rostock, Germany; 7 Department of Electrical and Computer Engineering, KIOS Research Center, University of Cyprus, Nicosia, Cyprus; 8 Medical Physics and Biomedical Engineering Department, University College London, London, UK; 9 Department of Natural Sciences, Middlesex University, London, UK; 10 Division of Neonatology and Pediatric Intensive Care, University Hospital of Geneva, University of Geneva, Geneva, Switzerland

**Keywords:** Paediatrics, Medical research

## Abstract

Endotracheal suctioning is a widely used procedure to remove secretions from the airways of ventilated patients. Despite its prevalence, regional effects of this maneuver have seldom been studied. In this study, we explore its effects on regional lung aeration in neonates and young infants using electrical impedance tomography (EIT) as part of the large EU-funded multicenter observational study CRADL. 200 neonates and young infants in intensive care units were monitored with EIT for up to 72 h. EIT parameters were calculated to detect changes in ventilation distribution, ventilation inhomogeneity and ventilation quantity on a breath-by-breath level 5–10 min before and after suctioning. The intratidal change in aeration over time was investigated by means of regional expiratory time constants calculated from all respiratory cycles using an innovative procedure and visualized by 2D maps of the thoracic cross-section. 344 tracheal suctioning events from 51 patients could be analyzed. They showed no or very small changes of EIT parameters, with a dorsal shift of the center of ventilation by 0.5% of the chest diameter and a 7% decrease of tidal impedance variation after suctioning. Regional time constants did not change significantly. Routine suctioning led to EIT-detectable but merely small changes of the ventilation distribution in this study population. While still a measure requiring further study, the time constant maps may help clinicians interpret ventilation mechanics in specific cases.

## Introduction

Chest electrical impedance tomography (EIT) is on its way from being a research tool to being applied to clinical problems^1^. Due to its radiation free operation and functional imaging capabilities at high scan rates of 50 frames per second or more, it is especially suited for monitoring neonates’ and young infants’ lungs at the bedside.

In the intensive care unit (ICU), suctioning is a common intervention which is expected to have an instantaneous effect on EIT findings, as has been shown in earlier works: Veenendaal et al. showed a lung volume loss during suction, followed by a fast and almost complete recovery in infants^2^. Interestingly, Hough et al. even observed an increase in lung volume for at least 90 min after the initial volume loss caused by suction^3^. Closed suctioning is generally considered the more protective suctioning system as it may prevent loss of lung volume, especially when paired with a smaller catheter^4^. On the other hand it was found that at least adults need longer to recover from lung volume loss after closed suctioning^5^.

The aim of suctioning is to remove fluids and mucus from the airways and thereby either to reopen occluded lung areas or to reduce the airway resistance. With passive expiration, the change of air volume in the lung may be described as

1 $$\begin{aligned} V(t) = V_0 \cdot \textrm{e}^{-\frac{t}{\tau }} + V_{\text {FRC}}, \end{aligned}$$

with *V*(*t*) being the lung volume at time *t*, starting with $t=0$ at the end of inspiration, $V_0$ being the lung volume at the start of expiration and $V_{\text {FRC}}$ being the lung volume at the end of expiration, corresponding to the functional residual capacity. The time constant $\tau$ is the product of respiratory system compliance $C_{rs}$ and airway resistance $R_{aw}$. Feasibility and reliability of EIT-derived time constants have been demonstrated in adult patients^6,7^. Miedema et al. calculated time constants in neonates after recruitment steps under high frequency oscillatory ventilation (HFOV), but not in individual breaths and for individual image pixels like in this work^8^.

In this study we investigate whether routine suctioning causes EIT-detectable changes in regional ventilation and respiratory system mechanics. We concentrate not on immediate suctioning effects which have been addressed previously but on possible later changes between five and ten minutes after the intervention. The information on the effects of suctioning might contribute to better assessment of its efficacy and improve the indications of this intervention, known to be associated with complications in a large proportion of patients^9^.

## Methods

### CRADL dataset

The Continuous Regional Analysis Device for neonate Lung (CRADL) project was an EU-funded project that included an observational clinical study in which EIT technology was applied to neonates and young infants in multiple ICUs across Europe (EU grant No. 668259)^10^. All methods were carried out in accordance with the relevant guidelines and regulations. All experimental protocols were approved by the local ethics committees and written informed consent was obtained from both parents. The respective ethics committees were Medisch Ethische Toetsingscommissie Amsterdam UMC (METC 2016/184), Cyprus National Bioethics Committee (EEBK/EP/2016/32), and Ethical Committee of the Northern Ostrobothnia Health Care District (EETTMK: 35/2017). With 200 patients included for up to 72 h of recording time it is one of the largest clinical study using EIT to date. The primary endpoint of this study was to demonstrate the feasibility of EIT monitoring in critically ill neonates and young infants. Secondary endpoints of this study considered the effects of numerous documented events, such as intubation/extubation, suctioning, or posture changes, on regional lung aeration as measured with EIT. As part of the clinical study of the CRADL project 60 terabytes of data were recorded, including clinical and demographic patient data, EIT measured voltages and reconstructed images, documented events and a low-framerate video log.

$\text {BB}^2$ EIT devices (Sentec AG, Landquart, Switzerland) were used in this study. The non-adhesive textile electrode belt comprising 32 electrodes with the dimensions 20 mm times 5 mm was placed along the 5th to 6th intercostal space^11^. Neonatal ultrasound gel (Aquasonic 100, Parker laboratories Inc Parker, NJ, USA) was used to improve the electrode-skin contact impedance. The device injects an alternating current with a frequency of 200 kHz and an amplitude of 3 $\text {mA}_\text {rms}$. Both current injection and sequential voltage measurement are done using a “skip 4” pattern. The device internal image reconstruction was used in this study: The recommendations of the Graz consensus Reconstruction algorithm for EIT (GREIT) publication were followed to construct a finite element model of the chest^12^. The shape was obtained by manually segmenting the CT scan of a single 14-months-old male (height 760 mm, weight 9500 g). Regularization was done according to the standard implementation of the GREIT algorithm in EIDORS^13^: As regularization matrix the identity matrix multiplied by a scalar was taken. The scalar was chosen so that the Noise Figure of 0.5 was achieved. Input parameters for GREIT were 0.05 for target size, 0.25 to draw the so-called desired image, and 1000 training points in a uniform distribution. All other parameters were set according to the GREIT paper or to the default implementation of GREIT in EIDORS. The reconstruction matrix was derived following the default implementation in EIDORS, which is based on a non-weighted L2-norm. To ensure consistency and comparable results, the same finite element model and regularization was applied for all patients and recordings, leading to the same reconstruction matrix for all patients and recordings. In accordance with the GREIT algorithm, no explicit priors were applied.

The image reconstruction workflow is not optimized for the reconstruction of oscillations over a certain threshold frequency, which is why we excluded patients on HFOV.

For this paper we analyzed all documented tracheal suctioning events in intubated patients included in the CRADL study.

### Tidal EIT parameters

During data acquisition, the events were documented by nurses at the bedside using a custom graphical user interface of the $\text {BB}^2$ EIT device. Naturally, these event marks vary in precision of both content and timing. To eliminate this variability, each suctioning event was validated using the video log. The events marked “suctioning” by the nurses include oral, nasal and tracheal suctioning maneuvers—only the latter were included. Using the video log we also discerned whether an open or closed suctioning system was used. We chose time intervals of 5 min length with a safety margin of 5 min before (baseline) and after the end of the validated event. This takes into account the usual signal disturbance by manipulation and consequent agitation of the patient surrounding the suctioning events. These intervals were then analyzed in the following fashion: first we applied a breath detection algorithm^14^ to identify the end of inspiration and expiration in the summed impedance signal. We used the difference between corresponding reconstructed images of inspiration and expiration to calculate tidal impedance variation images. Common functional EIT parameters were then calculated on the whole thoracic cross-section in these tidal images: center of ventilation right–left, center of ventilation ventral-dorsal, coefficient of variation, global inhomogeneity index and tidal impedance variation. For details about these parameters and their exact calculation we refer to^15^. We refrained from using functional or anatomical regions of interest because of the known problems and added complexity of these methods^16^.

### Expiratory time constants

We manually selected 30 s intervals of stable breathing within the aforementioned 5 min windows before and after the suctioning event. In some cases, the interval was selected from within the 5 min safety margin if it was evident that there was sufficient time to/from the actual event.

To determine the regularity and stability of the breathing pattern in the selected data sections, two quantitative measures characterizing both the respiratory rate and the tidal volume were computed: (1) the duration of each breathing cycle, and (2) the amplitude of each tidal impedance change. Subsequently the median values and interquartile ranges were calculated from these values within the whole 30-s intervals.

Analog to the change in volume over time (Eq. 1) we can model the change in impedance with an exponential function:

2 $$\begin{aligned} Z(t) = Z_0 \cdot \textrm{e}^{-\frac{t}{\tau }} + Z_{\text {res}}, \end{aligned}$$

with *Z*(*t*) being the raw pixel impedance signal (with respect to a reference image), *t* the time from the last end of inspiration and $\tau$ being the expiratory time constant. $Z_0$ is the positive, variable part of the impedance signal when considering expiratory time constants (negative for inspiratory time constants). $Z_{\text {res}}$ is the invariable part of the impedance signal caused by varying regional baseline impedances and the arbitrary impedance baseline.

Some lung areas reach their respective maximal and minimal aeration earlier than others. In order for the exponential regression to function accurately and reliably for every image pixel, we allowed for a certain amount of time shift of individual pixel minima/maxima with respect to the globally detected impedance minima/maxima. More precisely, we applied a low-pass filter to both the global impedance signal and each individual pixel impedance signal to remove high-frequency noise like cardiac oscillations. We used a 3rd order butterworth filter with a 2 Hz cutoff frequency. Considering one expiration in the global signal, we extended the beginning of the analysis interval by half the time from the preceding minimum. Similarly, the end of the interval was extended by half the time to the next maximum. Within this interval, we first discarded all data points before the first relative maximum and after the last relative minimum. We then discarded the top and bottom 10% of the data. This is a compromise between keeping the maximum amount of data and discarding parts that are known to be particularly noisy or unreliable, see also^17^. Finally, we performed the regression with the above exponential formula with the selected intervals but without low-pass filtering beforehand.

To produce pixel-wise time constant maps that are robust and less noisy, we aggregated 30 s worth of breaths into one map. Each pixel represents the median time constant if $R^2$ of the regressions was at least 0.8 in at least half of the globally detected breaths. To assess the variability of the calculated time constants within the 30 s interval, we also computed maps of interquartile ranges for every image pixel.

As numerical representations for the distributions of time constants within one map, we calculated the weighted mean time constant $\bar{\tau }_w$ and weighted mean standard deviation $\text {SD}_{\bar{\tau _w}}$ with the weights being proportional to the corresponding pixel values in the tidal image. These numerical representations were calculated on the whole thoracic cross-section. For this study, we defined a decrease of weighted mean time constant of at least 10% below baseline as an effective maneuver. This threshold only serves an illustrative purpose; it was not used to calculate statistical results.

### Statistical analysis

For the statistical analysis, we did not assume a normal distribution and thus used a paired Wilcoxon–Mann–Whitney-test. The significance level was 5%. To correct for multiple testing, we applied Bonferroni correction for the 10 statistical comparisons, resulting in a Bonferroni threshold of 0.5%.

## Results

804 events of 107 patients were documented during the measurement. 248 events were excluded because no suctioning event could be visually identified using the video log (missing video data, misplacement of camera, obscured view, incorrect documentation). 55 additional, previously undocumented events were identified during this step. 236 events were discarded because they included only oral or nasal suctioning. Further 31 events were excluded because the patients were ventilated under HFOV. This left us with 51 patients with a total of 344 events. Table 1 shows the demographic data of the whole study population and for the subgroup of those patients that could be included in this analysis.

**Table 1:** Descriptive statistics of study population (median (interquartile range)).

Parameter	Whole CRADL	Analysis of EIT parameters	Analysis of time constants
Patient count	200	51	11
Event count	375	344	71
Male:female	121:79	29:22	8:3
Postmenstrual age (weeks)	33.2 (6.0)	33.4 (7.0)	31.7 (5.5)
Weight (g)	2234 (1500)	2353 (1580)	1784 (1175)
Prematurity prevalence (%)	53	45	82
Respiratory distress syndrome prevalence (%)	39	76	91
Pneumonia prevalence (%)	4	6	0

### Tidal EIT parameters

**Figure 1:**
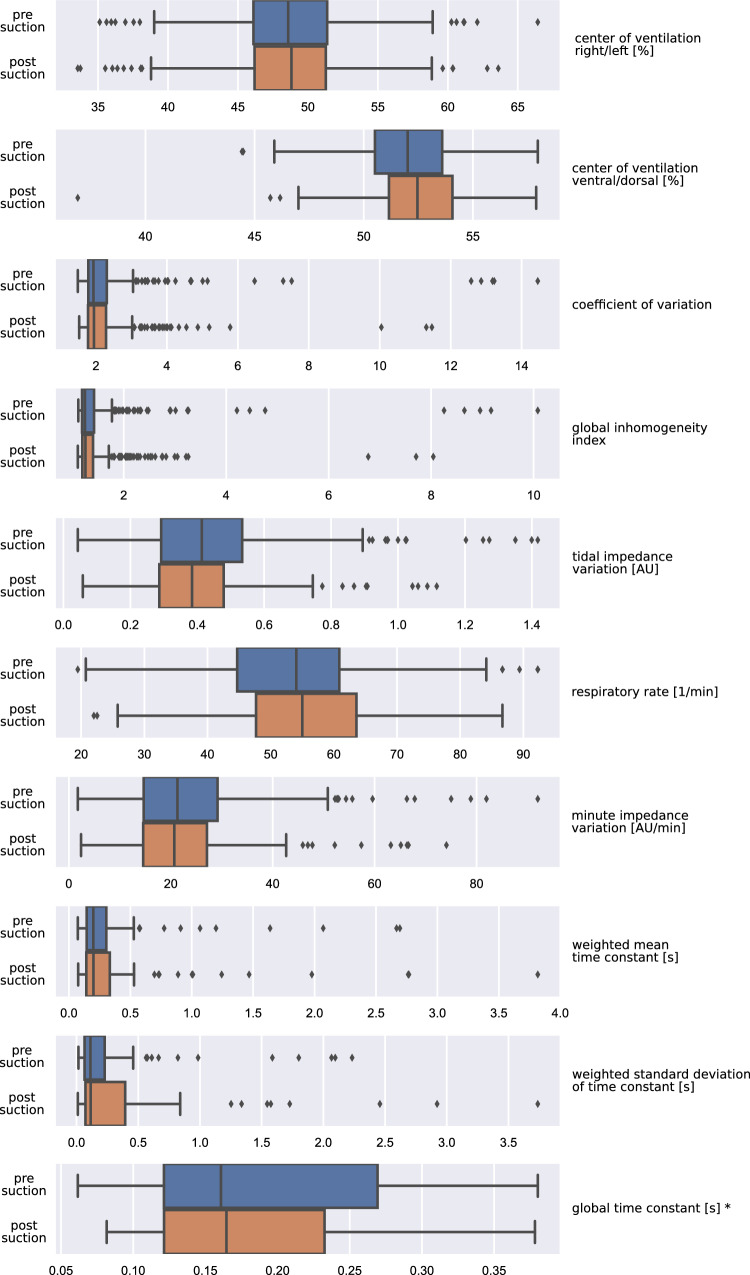
Box plots of all results derived from the EIT measurements before and after endotracheal suctioning (for summary results see also Tables 2 and 3). *Outliers removed.

Table 2 shows the calculated EIT derived parameters before and after suction as well as the corresponding statistical analysis. We observed a significant dorsal shift of the center of ventilation and a 7% decrease in the tidal impedance variation; all other EIT parameters did not change significantly. Although the respiratory rate rose significantly, the minute impedance variation (product of tidal impedance variation and respiratory rate) did not change significantly. Box plots of the calculated tidal EIT parameters are presented in Fig. 1.

**Table 2:** EIT derived tidal parameters and respiratory rate before and after suction (344 events) (median (interquartile range)).

Parameter	Before	After	p-value
Center of ventilation right/left (%)	48.59 (5.28)	48.82 (5.18)	0.83
Center of ventilation ventral/dorsal (%)	52.01 (3.08)	52.46 (2.91)	<** 0.001**
Coefficient of variation	1.93 (0.523)	1.94 (0.509)	0.55
Global inhomogeneity index	1.24 (0.236)	1.25 (0.210)	0.73
Tidal impedance variation [AU]	0.414 (0.242)	0.384 (0.192)	<** 0.001**
Respiratory rate ($\hbox {min}^{-1}$)	54.05 (16.17)	55.02 (15.89)	<** 0.001**
Minute impedance variation [AU/min]	21.3 (14.5)	20.7 (12.5)	0.038

*AU* arbitrary units.

Significant values are in bold.

### Expiratory time constants

**Figure 2:**
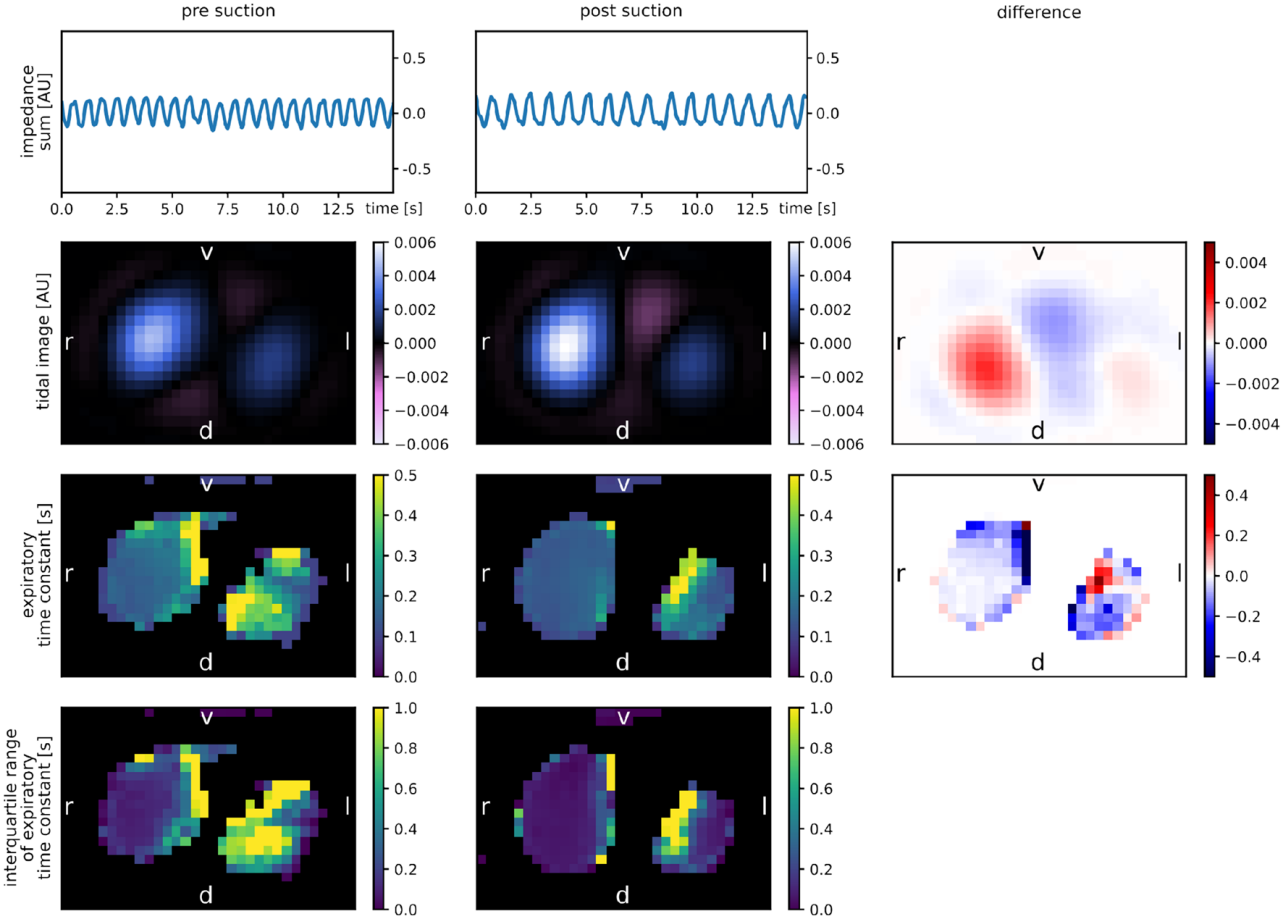
Maps of calculated values of respiratory mechanics (in rows: time trace of sum impedance signal, tidal image, expiratory time constants, interquartile range of expiratory time constants; in columns: pre suction, post suction, difference). All maps are axial slices seen from below with the patient’s back at the bottom of the map (DICOM orientation). Patient is a premature male, body weight 3000 g, post menstrual age 36 weeks treated for respiratory distress syndrome. *r* right, *l* left, *v* ventral, *d* dorsal.

A subgroup of 71 events of 11 patients with especially regular breathing patterns could be included in this analysis. The median duration of one breathing cycle (and interquartile range) was 0.975 s (0.160 s) and the median summed tidal impedance variation was 0.380 AU (0.091 AU). Example excerpts from the summed impedance signals used for the calculation of expiratory time constants before and after suction are shown in the first row of Fig. 2.

Figure 2 shows examples of maps of expiratory time constants before and after suctioning events. In the second row, the tidal images before and after suction and their difference are presented. Areas of high ventilation, plotted in blue, correspond to the left and right lung region. In between, there are small purple areas with out-of-phase impedance changes, probably due to artifacts at the lung-heart boundary^18^. An increase in ventilation is noted in the right lung (dark blue area in the difference image, second row, last column). In the third row, all pixel time constants that could be calculated according to the specified quality criteria are visualized on a black background. Large time constants are present in ventromedial parts of the right lung (yellow, third row, first column) which do not persist after suction. As a quality indicator, the fourth row shows the interquartile range of all expiratory time constants that were calculated for one image pixel. Here, the variability is low in well-ventilated areas and high in the medial parts of both lungs (as well as in the dorsal part of the left lung in the “pre suction” image).

Table 3 shows different parameters derived from the exponential regression and the corresponding statistics. The observed parameters derived from the exponential regression did not change significantly after suctioning. Box plots of these values are shown in the bottom part of Fig. 1.

**Table 3:** Parameters derived from exponential regression (71 events) (median (interquartile range)).

Parameter	Before	After	p-value
Weighted mean time constant (s)	0.199 (0.159)	0.200 (0.191)	0.68
Weighted standard deviation time constant (s)	0.113 (0.163)	0.115 (0.323)	0.32
Global time constant (s)	0.161 (0.147)	0.165 (0.111)	0.77

As described earlier, we defined a decrease of weighted mean time constant of at least 10% below baseline as an effective maneuver. According to this criterion, 38% of the performed maneuvers were effective.

## Discussion

In this study we analyzed 344 routine tracheal suctioning maneuvers in ventilated neonateal and pediatric patients with EIT. We calculated common tidal EIT parameters and regional time constants.

For the tidal parameters we relied on parameters that are well established and that are considered useful by clinicians^19^. These tidal parameters only take into account the difference between EIT images at the end of inspiration and at the end of expiration—all data in between is ignored. The tidal parameters we analyzed in this work fall into three categories: measures of ventilation position (center of ventilation), measures of ventilation homogeneity (coefficient of variation and global inhomogeneity index) and measures of ventilation quantity (tidal impedance variation). The respiratory rate was also listed as an EIT parameter because in this case it was solely derived from the EIT signal.

If suctioning was successful at improving ventilation mechanics, one would expect to see EIT-detectable changes after the maneuver. In fact we see only small changes of the center of ventilation and the tidal impedance variation. The observed dorsal shift of the center of ventilation has been detected by others before^3^. It may be either explained by an increase of compliance in the dorsal lung areas or a decrease of compliance in the ventral lung areas. The change, although statistically significant, is extremely small: the center of ventilation moves by less than 0.5% of the ventrodorsal thorax diameter. The clinical relevance of this small change is uncertain. Overall, our results concerning the ventilation homogeneity imply no or only minimal changes associated with the suctioning events. Theoretically, one might hypothesize that removing secretions from (partly) occluded lung areas would increase ventilation in those areas whereas already well-ventilated areas would be mostly unaffected, resulting in a more homogeneous ventilation distribution. However, this was not observed. Another explanation for the unaffected global inhomogeneity index and coefficient of variation could be, that the suctioning catheters did not reach beyond the trachea and main bronchi, leaving occluded smaller bronchi unaffected. The reduced tidal impedance variation could be an effect of agitation, as the patients in this study are usually not deeply sedated. While tidal volumes decrease, the respiratory rate increases, resulting in an unchanged respiratory minute volume.

In this study we did not concentrate on the immediate short term effects of suctioning as others did. Therefore, we did not replicate the effects during and immediately after suctioning, especially the (contradicting) changes in end-expiratory lung impedance observed by others^2,3^. Young infants were shown to recover to baseline in about 8 seconds^2^, leaving only long-term effects visible 5 minutes later.

What we studied here were routine maneuvers which do not necessarily follow a common procedure as they are performed in different institutions by different individuals with different material. Especially suction pressures and the number and depth of catheter passes was not standardized, possibly resulting in weaker effects than suction under study conditions. Along the same line, patients in this study were treated for different diagnoses which may react differently to suctioning. However, we decided against performing additional tests for diagnosis subgroups because we have too little data and thus too small groups for meaningful statistical tests. Additionally, compared to suction in adults with lung injury, PEEP in neonates and young infants is usually low and catheter sizes are small. These factors make a pressure loss during the suction maneuver less critical and less likely, explaining why the observed changes are relatively small.

All parameters derived from tidal EIT images as well as from regional time constants were calculated for the whole thoracic cross-section because of the simplicity and robustness of this approach. All methods to select specific lung contours (or *regions of interest*) come with significant drawbacks^16^. Most prominently, individual anatomical lung contours would require harmful CT imaging (see below) and functional lung contours fail to include unventilated lung areas^20^.

Respiratory time constants are part of a physical lung model, which has been used for decades to help understand and explain respiratory mechanics^21^. Time constant calculation on an EIT pixel level has been done before, however only in adults^6,7^. What makes the time constant maps in this work special is the fact that they were calculated during regular ventilation (without forced maneuvers or airway pressure steps) in neonates with their high respiratory rate. The short duration of respiratory cycles results in few data points being available for every individual regression.

Since the time constant is the product of compliance and airway resistance, changes in the time constant cannot be attributed individually to changes of either value. In the context of an effective suctioning maneuver, one can hypothesize different effects: negative (sub-PEEP) pressure leads to local lung collapse and decreases local compliance, removal of obstructions reopens occluded lung areas and increases local compliance and/or also reduces airway resistance. In patients with variable amounts of spontaneous breathing effort, there is no way to discern changes in compliance from changes in airway resistance.

For the time constant analysis, we used intervals of 30 s because they represent a good compromise between feasibility of finding sufficiently long periods of stable breathing and robustness of the analysis. The quantitative evaluation of the breath duration and depth confirmed the regularity and stability of the breathing pattern during the analyzed periods. This was an important prerequisite for reliable calculations of median time constants of multiple breaths (averaged over the 30-s periods).

The weighted mean time constant correlates well visually with the time constant calculated from the global (summed) EIT signal. One of the disadvantages of the global time constant is the high number of outliers that occur with a signal to noise ratio as low as we have in this study. This is evident from the range of values: 0.062–32.47 s for global time constants versus 0.072–3.82 s for weighted mean time constants. This is why we chose the weighted mean time constant as our main numeric parameter derived from the exponential regression.

It is important to note that time constant calculation only works reliably for passive air flow, that is in ventilated patients without a relevant spontaneous breathing effort (during expiration). With increasing muscular activity of the patient, the flow profile usually becomes more constant which leads to a drastic overestimation of time constants. This is especially true for inspiration. One has to be cautious when interpreting these time constants since the detection of small amounts of spontaneous breathing effort is no trivial task.

However, there exists indirect evidence that the expiratory time constants that were calculated in this work are valid and useful. Firstly, we only analyzed the expiratory and not the inspiratory limbs of the EIT waveforms, where the effect of possible spontaneous breathing efforts are expected to be more pronounced. Secondly, breathing was relatively regular, the expiration was well-modelled by the exponential function and the resulting time constants were in a plausible range^6^. Thirdly, even if the patients exhibited relevant spontaneous breathing efforts, the methods applied in this study would still be useful to compare relative flows in different lung regions or at different times.

Unfortunately, within this study no clinical measure of success of the suction was recorded. As an example, oxygen saturation as measured with pulse oximetry would have been a useful addition to discern *clinically successful* suctioning maneuvers from those with no or negative effect. With an additional information like that, criteria or an algorithm to predict necessity of suctioning and expected suctioning success could be developed. The reason for this shortcoming lies in the source of the data: this study is a secondary analysis of data from the clinical trial of the CRADL project. The aim of the original trial was to demonstrate the feasibility of long-term EIT measurements in a critically ill neonatal/infant population routinely treated in neonatal/pediatric ICUs. To reach this already ambitious aim, no additional complexity in the form of other measurement devices and interventions was added.

CT imaging is rarely used in neonates because of the known high risks of tissue damage associated with radiation exposure^22,23^. That is why we had to use a single CT-based chest shape instead of individual chest shapes for the finite element model that is necessary for the EIT image reconstruction.

As stated earlier, some of the patients in the study population were under HFOV. These patients had to be excluded from the analysis because both our image reconstruction and breath detection are not well suited for this case.

Due to technical limitations in our setup, absolute impedance values (here end-expiratory lung impedance) may not be reliable across longer time periods if compensation events (electrodes losing/gaining contact) occurred in the meantime. Every time the electrode configuration changes, a different reconstruction matrix has to be used which may result in changes of the calculated absolute impedance values. In this kind of exploratory study setting, these compensation events occur quite often, which is why we did not include the affected values in our results.

## Conclusion

We studied the effects of endotracheal suctioning in critically ill ventilated neonates and young infants in one of the largest clinical EIT studies so far. We found that routine suctioning causes only very small changes in the ventilation distribution in the analyzed time window. Systematic effects on the distribution of respiratory time constants could not be identified. The absence of larger changes in regional ventilation and respiratory mechanics after suctioning implies that the usefulness of this regular clinical intervention might need to be re-evaluated. Specific patient groups potentially benefiting from the procedure might need to be clearly identified. More research, especially with respect to clinical success of suctioning with added reference monitoring methods, is necessary.

## Data Availability

The data used in this work stems from the CRADL clinical trial and is not publically available. Our ethics commitees do not allow consent to publish the raw data.
